# An *ex vivo* model using human peritoneum to explore mesh-tissue integration

**DOI:** 10.1242/bio.024992

**Published:** 2017-07-31

**Authors:** Peter Falk, Fernando Ruiz-Jasbon, Karin Strigård, Ulf Gunnarsson, Marie-Lois Ivarsson

**Affiliations:** 1 Department of Surgery, Institute of Clinical Sciences, Sahlgrenska Academy at University of Gothenburg, SE-416 85 Göteborg, Sweden; 2 Department of Surgery, Hallands Hospital, SE- 434 80 Kungsbacka, Sweden; 3 Department of Surgical and Perioperative Sciences, Umeå University, SE-901 85 Umeå, Sweden

**Keywords:** Experimental model, Peritoneum, Peritoneal tissue, Mesh, Synthetic mesh, Biocompatibility

## Abstract

Biological compatibility, in terms of implantation of foreign mesh material in hernia surgery, still needs experimental investigation. The present study develops an experimental model using human peritoneum to study the integration between tissue and different mesh material. The *ex vivo* model using peritoneal tissue was studied with different mesh material, and integration was monitored over time using microscopy. The peritoneal model could be kept viable in culture for several weeks. Cell migration was seen after 7-10 days in culture and could be further monitored over several weeks. The use of a human artificial model environment enabling the investigation of tissue/mesh integration has, to our knowledge, not been described previously. This proof-of-concept model was developed for the investigation of peritoneal biology and the integration between tissue and different mesh material. It has the potential to be useful in studies on other important biological mechanisms involving the peritoneum.

## Abstract (precis)

**Summary:** This study developed a human experimental model for long-term studies using peritoneal tissue to evaluate integration with different materials, such as synthetic meshes.

## INTRODUCTION

Most surgical repairs include use of meshes of different material, thereby introducing foreign material into the surgical field. It is well known that some meshes give rise to capsule formation, while others are integrated into the surrounding connective tissue. Our knowledge about biological integration of meshes in terms of compatibility, tissue remodeling and repair is insufficient. One reason for this is the lack of human experimental models.

The peritoneum is a membrane covering an area of approximately 2 m^2^ in an adult person (DiZerega, 2000; van der Wal and Jeekel, 2007), and has been the subject of investigation for decades. Our understanding of peritoneal healing and postoperative adhesion development due to altered fibrinolytic capacity is crucial, and has previously been investigated using cultured mesothelial cells (Falk et al., 2013; Mutsaers and Wilkosz, 2007). It is unlikely that an experimental model using a monolayer of mesothelial cells is representative of the peritoneum in the surgical situation, since certain factors are missing or vary, in this case the connective tissue and source of the cultured mesothelial cells. Healing and adaptation after surgery are complex mechanisms involving biological factors that are not fully understood. A preparation of intact peritoneal tissue, including the mesothelial layer and surrounding connective tissue with capillaries and lymphatic structures, might be a better alternative for the investigation of biological processes involving the peritoneum than a monolayer of cultured cells.

The role of the peritoneum is central, and studies on peritoneal healing include not only aspects on tissue-mesh integration, but also the role of different surgical techniques, different mesh materials and the formation of adhesions. A human experimental *ex vivo* model might have the potential to facilitate further studies on these issues.

In the present study, we demonstrate an experimental *ex vivo* model using human peritoneal tissue to investigate the integration between human tissue and different prosthetic materials.

## RESULTS AND DISCUSSION

### The *ex vivo* model

**Fig. 1.**
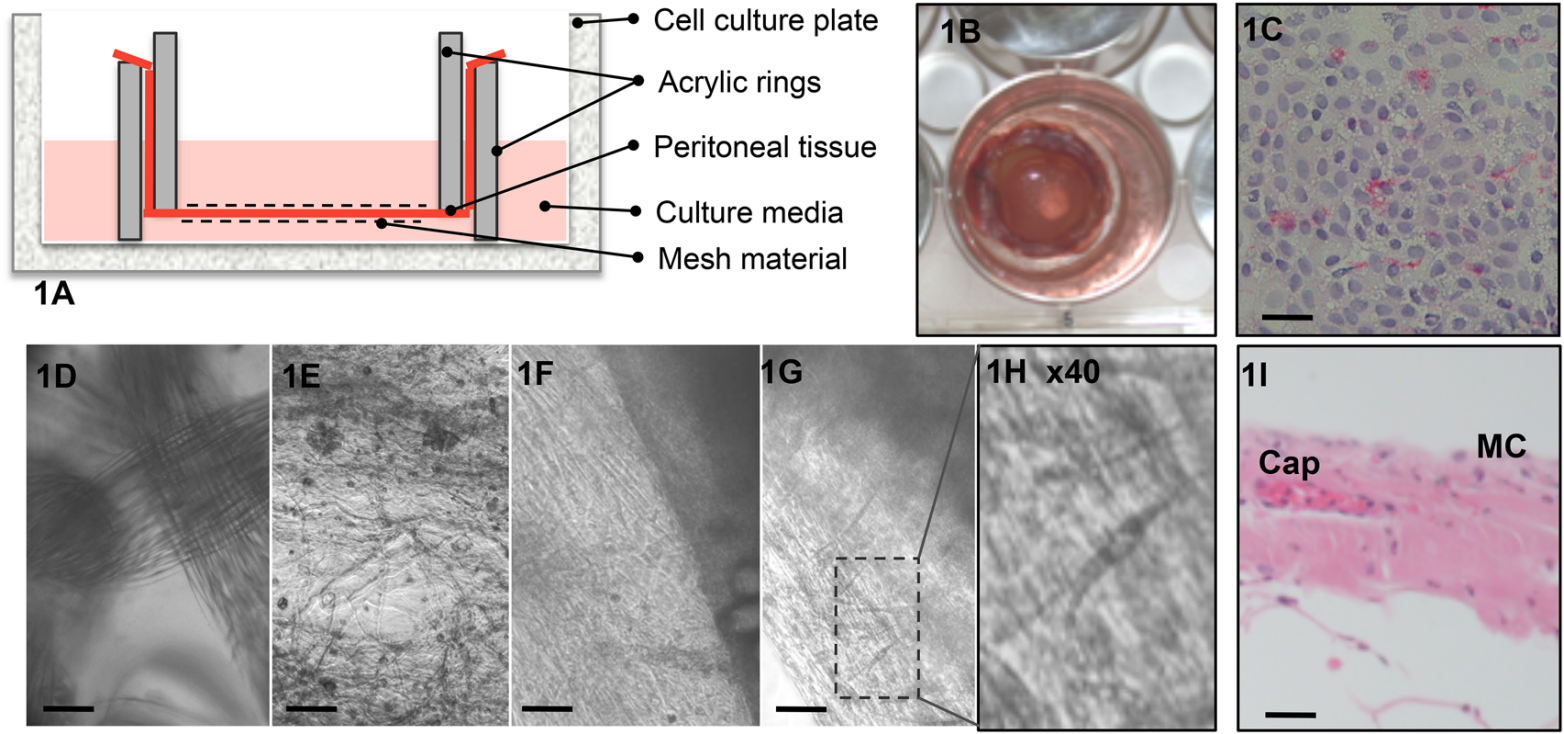
**The *ex vivo* model using human peritoneal tissue.** During sterile conditions the peritoneal tissue is fitted between two acrylic rings (A) and placed into a cell culture well, enabling an inside and an outside of the peritoneal membrane (B). An imprint of the peritoneal tissue using glass slides confirms the present layer of mesothelial cells using Hematoxylin/Eosin staining of an ‘en face’ *häutchen* preparation (C). Using inverted light microscopy, the peritoneal membrane could be monitored and documented with (D) and without (E) the presence of a mesh. After 7-10 days in culture, cells in the peritoneal tissue start to migrate to the foreign synthetic mesh (Bard Soft) (G,H). These migrating cells could not be seen at Day 3 in the *ex vivo* model (F). Histology confirmed several layers of the peritoneal tissue used (I). MC, mesothelial layer; Cap, capillary with endothelial cells. Scale bars: 100 µm.

In the present study, we were able to demonstrate *ex vivo* cultures of adult human peritoneal tissue that remained viable for several weeks under controlled laboratory conditions (Fig. 1). To our knowledge this has not been achieved previously. This provides new and improved possibilities for studying biological interactions between peritoneum and different foreign materials used for implantation in novel and future surgical procedures.

By using inverted microscopy, the peritoneal tissue could be monitored and documented with and without the presence of different meshes (Fig. 1D,E). The peritoneal tissue remained viable >26 days and in most cases >30 days in culture, showing migrating peritoneal cells (Fig. 1F-H). Daily observation verified that there was no microscopic contamination in the culture medium and no signs of bacteria present in the bottom of the culture wells. Moreover, samples of medium taken for the detection of bacterial growth at Days 1 and 26 revealed no contamination.

In general, there is a lack of human experimental models focusing on the integration of implanted foreign mesh used in hernia surgery into the tissues of the human abdominal wall. Until now, most research has been conducted using experimental models in animals. Cobb et al. presented a study on the strength of the abdominal wall after ventral hernia repair using different forms of polypropylene mesh in a porcine model (Cobb et al., 2006). Dolce et al. investigated the formation of adhesions to different composite mesh material in a rabbit ventral hernia model (Dolce et al., 2012). A hernia model in rabbits was also used by Yeo et al. (2014), who further investigated the effectiveness of a synthetic bioabsorbable scaffold. In a clinical study on giant hernia in 2010, Johansson et al. concluded that the postoperative strength of the abdominal wall muscles did not depend on the technique used for repair, and that the choice of surgical technique should be directed by anatomical circumstances (Johansson et al., 2011). However, the integration between tissue and implanted mesh material was not investigated in that study.

The use of dissected peritoneal tissue as an experimental model for studying the role of the peritoneum in gastric tumor cell dissemination in the abdominal cavity has been described previously (Cabourne et al., 2010). In that study, Cabourne et al. could not keep their peritoneal tissue viable for >3-4 days in culture. However, in the present study we observed that viable cells continued to migrate into the peritoneal tissue after several weeks in culture. We cannot fully explain the reason for the differences between our results and those of Cabourne et al. However, there are differences in the handling of the peritoneal tissue because Cabourne et al. used sodium chloride as a transport medium from the operation theater to the laboratory (Cabourne et al., 2010), whereas culture medium at normal pH was used in the present study. The use of sodium chloride in surgery for rinsing the abdominal cavity has been the subject of discussion because it has a low pH if not buffered (Połubinska et al., 2006) and has also been considered cytotoxic in models using cultured cells (Połubinska et al., 2008). These differences may be of limited significance but should be noted.

Peritoneal healing and repair has been described for many years but only in recent decades has our understanding of the mechanisms regulating this process become more established. The principles of mesothelial remodeling and regeneration differ from the repair of traditional wound healing (Mutsaers et al., 2007; Mutsaers, 2002). Mesothelial differentiation is one important part in peritoneal remodeling that occurs even in culture under certain conditions such as stimulation by growth factors (Yang et al., 2003; Falk et al., 2013; Falk et al., 2008). When peritoneal tissue is cultured over a longer period of time it is likely that there is a certain progress of differentiation in the cultured cells. Cell differentiation might have taken place in the present study, as indicated by the structural changes in peritoneal tissue seen over time, but this was not further investigated.

Peritoneal healing and mechanisms for development of adhesions are crucial after abdominal surgery. Due to differences in fibrinolytic capacity resulting in an imbalance in fibrin formation and fibrin degradation, there are possibilities for creating postoperative adhesions (Falk et al., 2013; Mutsaers and Wilkosz, 2007). Some of the involved factors present in the peritoneal tissue might be possible to further investigate using a peritoneal *ex vivo* model, in contrast to culture mesothelial cells only.

Furthermore, not to jeopardize the healing properties of the donor's parietal peritoneum, thereby introducing an ethical dilemma, the peritoneal tissue used in the present study was taken from the part of the inguinal hernia sac that had been removed. *In vivo* data support the notion that mesothelial proteins secreted at different anatomical sites are similar (Serre et al., 2003) but healing properties may vary. Also, when obtaining peritoneal samples, gentle handling with sharp dissection, without electrocoagulation or other energy emitting devices, was employed. Histology specimens of the sampled peritoneal tissue confirmed several layers in the tissue, including mesothelial cells and visible capillaries (Fig. 1I). Despite this, tissue with an intact mesothelial surface and totally free from damage from the surgical procedure could not be guaranteed. This also applies to the stress-affected part of the peritoneum and possible damage to phospholipid layers present in the mesothelial cell membrane, such as hyaluronic acid or glycosaminoglycans (Mutsaers and Wilkosz, 2007).

### *Ex vivo* model in the presence of synthetic mesh

**Fig. 2.**
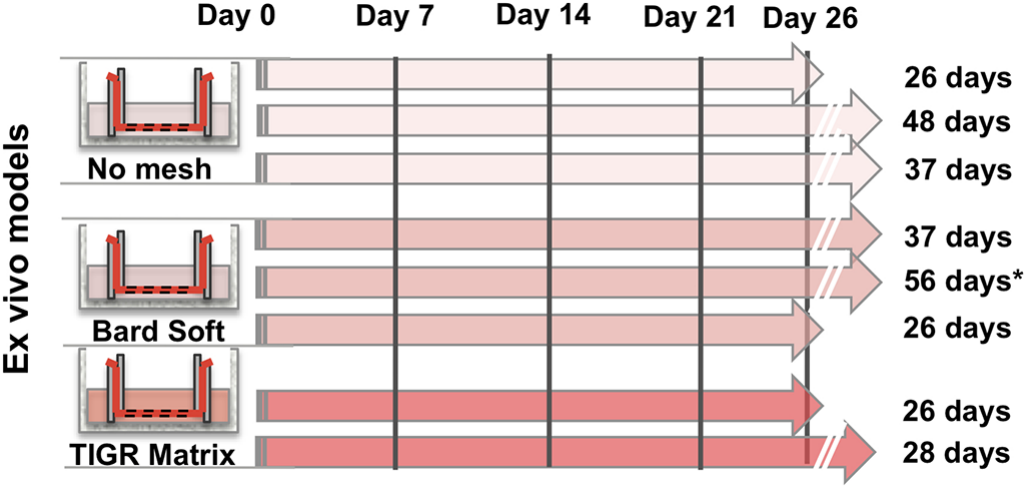
**Summary of performed experiments.** Together with the peritoneal tissue alone, two different synthetic meshes (Bard Soft and TIGR Matrix) were investigated for ≥26 days. Each arrow indicates a duplicate of experimental set-ups (*n*=2-3) of *ex vivo* models (*n*=4-6 for each group). The asterisk indicates one (of two) set-ups that was discarded after 60 days in culture due to possible ocular bacterial contamination. Samples from the conditioned culture media revealed no bacterial contamination.

**Fig. 3.**
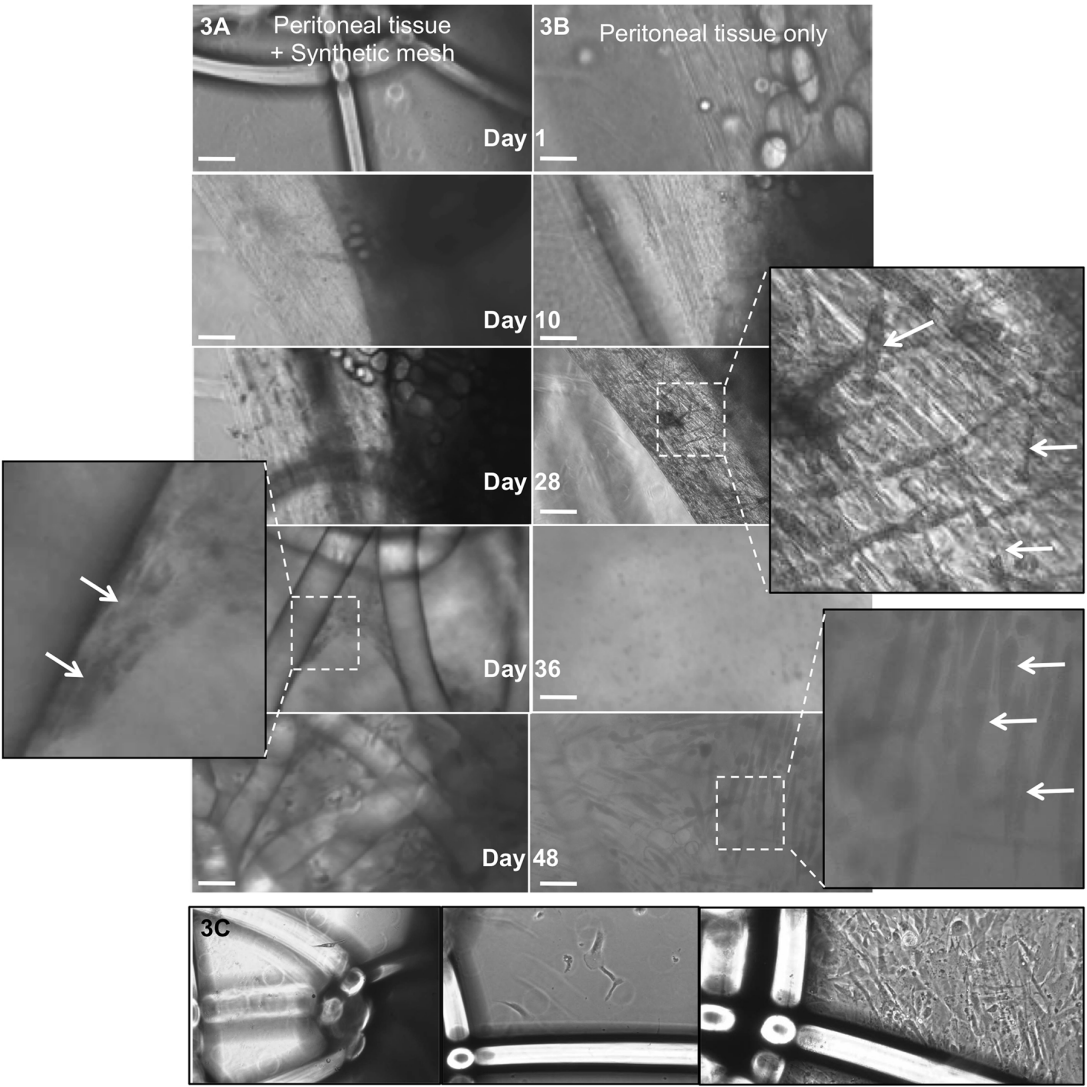
**Time lapse of the *ex vivo* peritoneal tissue model during culture, with and without the presence of a synthetic mesh.** The peritoneal tissue could be kept in culture ≤56 days without bacterial contamination. Cells migrating from the peritoneal tissue could be monitored from Day 10 onwards (arrows). No differences in cell migration or cell count were seen between experimental set-ups with or without the presence of the synthetic mesh (A,B). Migrating cells found in the bottom of the chamber/well were identified as fibroblasts by their growth and morphological characteristics (C). Scale bars: 100 µm.

When synthetic mesh was incubated together with the peritoneal tissue, as illustrated (Fig. 2 and Fig. 3A,B), all the *ex vivo* models with a mesh could be kept in culture for 26 days, and some were kept for 56 days without any sign of bacterial contamination (Fig. 2). Further experiments, including monitoring and photographic documentation, were performed using Bard Soft Mesh. Time-lapse analysis of the *ex vivo* model with or without mesh, was used to monitor cell coverage over time. The primary coverage of cells from the peritoneal tissue and adaptation into the mesh could be seen after approximately 1 week (Day 7). Single cells began to migrate towards the foreign material and this escalated with time (Fig. 3A,B).

Between Days 17 and 28 more activity was seen in the model, illustrated by an increase in cell count and amount of migrating cells. By Day 48 large areas of the synthetic mesh were covered with fibroblasts migrating from the peritoneal tissue.

Migrating cells finally found in the cell culture bottom were later verified as fibroblasts due to their morphological appearance and growth characteristics (Fig. 3C). No differences in cell count, cell activity or viability were observed between the *ex vivo* models with and without synthetic mesh, nor were any differences seen between the models with the two different types of mesh.

Sotiri et al. reviewed the problems concerning adhesion to foreign materials used in medicine by investigating the use of immobilized liquid layers as an approach to decrease tissue adhesion to medical devices (Sotiri et al., 2016). In order to further understand the integration between tissue and foreign materials, Whelove et al. (2011) presented a new technique adding nanoparticles covered with gold to improve biocompatibility (Whelove et al., 2011). They used nanoparticle-covered polyethylene mesh in their *in vitro* experiments, showing improved adhesion of L929 fibroblasts and decreased bacterial adhesion to the synthetic mesh. The fact that their experimental model was an *in vitro* rather than an *ex vivo* model meant that a simple monolayer of cultured cells was used as opposed to an intact part of an organ, as in the present study using peritoneum.

In the present experimental *ex vivo* model, two types of synthetic meshes were used: one synthetic nonabsorbable and one synthetic absorbable version. The main requisite is that the mesh must be semi-transparent to enable transmission of light using inverted microscopy. In future experimental models comparisons will be performed between other types of mesh, both synthetic and biological. Other types of visualization systems will then be used.

In summary, we have developed a proof-of-concept model of adult human peritoneal tissue, for the investigation of peritoneal biology and the incorporation of foreign mesh material. *Ex vivo* peritoneal preparations could be kept viable under controlled laboratory conditions for up to 56 days, and this, to our knowledge, has not previously been described.

The present *ex vivo* model has the potential to become an important research tool for studies on the peritoneum or areas of research in which the peritoneal membrane is a key component. Furthermore, this model provides new possibilities for studying biological integration between tissue and potential novel materials for implantation in future surgical procedures, in humans and other mammals.

## MATERIALS AND METHODS

If not otherwise stated, all chemicals and cell culture reagents were purchased from Sigma-Aldrich (St Louis, USA).

### The *ex vivo* peritoneal model

After having gained informed consent, peritoneal tissue was isolated, from five patients undergoing routine surgery for inguinal hernia repair. Fresh tissue samples were placed in a sterile bowl and submerged in sterile culture medium E199. Within 5 min the sterile bowl was transported from the operation theatre to the laboratory, where the tissue was immediately dissected to remove extra-peritoneal fat. The samples were then cut into squares approximately 25×25 mm to be mounted in the *ex vivo* model as described (Fig. 1). Depending on variations in the size of the peritoneal tissue extracted, different numbers of experimental set-ups were obtained. A total of eight experimental set-ups were performed (three without mesh, three with Bard Soft Mesh and two with TIGR Matrix, with each set-up performed as a duplicate) (Fig. 2). Carefully avoiding damage to the mesothelial surface, the tissue was gently suspended between two acrylic rings with the mesothelial cell surface pointing upwards, and submerged in culture medium in a six-well culture dish without touching the cell culture plate. Culture medium (E199) supplemented with 30 IU/ml penicillin and streptomycin (PEST), 1.1 mM L-glutamine, 20% fetal calf serum (FCS), 0.5 µg/ml hydrocortisone, 50 µg/ml growth factor according to Maciag et al., (1979) and 10 IU/ml heparin (Leo Pharma, Malmö, Sweden) was used as described previously (Reijnen et al., 2001; Falk et al., 2013). The *ex vivo* model specimens were cultured in a CO_2_ incubator (Forma, Ninolab, Upplands Väsby, Sweden) at 37°C with 5% carbon dioxide. The culture medium was completely changed three times a week (Fig. 1).

All handling in the cell culture laboratory was performed under sterile conditions in the area of a laminar-air-flow (LAF) unit (Holten, Ninolab, Upplands Väsby, Sweden). Monitoring of changes in the experimental model was performed using inverted phase-contrast microscopy and photographic documentation (Axiovert 25 and Axiovision, Carl-Zeiss Gmbh, Jena, Germany). Repeated photographic documentations of the experimental set-ups were performed individually and consecutively every second or third day until each set-up was completed. Each experiment was performed in duplicate in two or three independent experiments.

### Viability of peritoneal tissue and bacterial contamination

Regular monitoring of the *ex vivo* specimens was performed, including the observation of any living cells being exfoliated from the isolated peritoneal tissue. No sign of contamination was seen in the culture medium or tissue during the first days of culture in the *ex vivo* model. Samples were also taken for detection of bacterial contamination of the medium at Days 1 and 26. Monitoring and photographic documentation were performed using inverted microscopy and the Axiovision system (Carl-Zeiss).

### Using the *ex vivo* model in the presence of synthetic mesh

In order to investigate integration between peritoneal tissue and foreign material, different types of mesh were placed on both the inside (mesothelial side) and outside (retroperitoneal side) of the peritoneal preparations produced in the *ex vivo* model described above. A large-pore monofilament polypropylene mesh (Bard Soft Mesh, Bard Davol, Warwick, USA) and an absorbable synthetic mesh (TIGR Matrix/Surgical Mesh, Novus Scientific, Uppsala, Sweden) were investigated.

### Ethics

The sampling of peritoneal tissue was conducted with the human patients’ understanding and consent, and was approved by the Local Ethics Committee (University of Gothenburg, Dnr Ö728-03).
